# Development and characterization of a new human hepatic cell line

**DOI:** 10.17179/excli2015-424

**Published:** 2015-07-28

**Authors:** Eva Ramboer, Bram De Craene, Joey De Kock, Geert Berx, Vera Rogiers, Tamara Vanhaecke, Mathieu Vinken

**Affiliations:** 1 In Vitro Toxicology and Dermato-Cosmetology research group, Center for Pharmaceutical Research, Vrije Universiteit Brussel, Laarbeeklaan 103, 1090 Brussel, Belgium; 2 Unit of Molecular and Cellular Oncology, Inflammation Research Center, VIB, Technologiepark 927, 9052 Zwijnaarde, Belgium; 3 Department of Biomedical Molecular Biology, Ghent University, 9052 Ghent, Belgium

**Keywords:** human hepatocytes, immortalization, hepatic cell line

## Abstract

The increasing demand and hampered use of primary human hepatocytes for research purposes have urged scientists to search for alternative cell sources, such as immortalized hepatic cell lines. The aim of this study was to develop a human hepatic cell line using the combined overexpression of TERT and the cell cycle regulators cyclin D1 and mutant isoform CDK4R24C. Following transduction of adult human primary hepatocytes with the selected immortalization genes, cell growth was triggered and a cell line was established. When cultured under appropriate conditions, the cell line expressed several hepatocytic markers and liver-enriched transcription factors at the transcriptional and/or translational level, secreted liver-specific proteins and showed glycogen deposition. These results suggest that the immortalization strategy applied to primary human hepatocytes could generate a novel hepatic cell line that seems to retain some key hepatic characteristics.

## Abbreviations

A1AT, alpha-1-antitrypsin; ACTB, beta actin; AFP, alpha-fetoprotein; ALB, albumin; B2M, beta-2 microglobulin; BSA, bovine serum albumin; BSEP, bile salt export pump; C/EBPα , CCAAT/enhancer binding protein α; CDK4, cyclin dependent kinase 4; CK, cytokeratin; Cy, cyanine; CYP450, cytochrome P450 monooxygenase; cDNA, complementary deoxyribonucleic acid; DIHH-ET cells, differentiated IHH-ET cells; ELISA, enzyme-linked immunosorbent assay; FITC, fluorescein; FSC, forward scatter; GAPDH, glyceraldehyde-3-phosphate dehydrogenase; G6PD, glucose-6-phosphate dehydrogenase; GGT1, gamma-glutamyltransferase 1; HEK293t, Human Embryonic Kidney 293t; HH, human hepatocytes; HNF, hepatocyte nuclear factor; HPRT1, hypoxanthine ribosyltransferase 1; IHH-ET cells, immortalized human hepatocytes; (m)RNA, (messenger)ribonucleic acid; MRP, multidrug resistance-associated protein; NC, negative control; NTCP, sodium-dependent taurocholate cotransporting polypeptide; PAS, Periodic acid Schiff; PBS, phosphate buffered saline; PC, positive control; PDT, population doubling time; PerCP, Peridinin chlorophyll protein complex; RIPA, radioimmunoprecipitation assay; R-PE, R-phycoerythrin; RT-qPCR, real-time quantitative polymerase chain reaction; SSC, side scatter; TBS-T, Tris-buffered saline solution; TERT, telomerase reverse transcriptase; UBC, ubiquitin C; WB, western blot.

## Introduction

Pharmaco-toxicological research of new chemical and biological entities is usually performed in experimental animals. Because of scientific, ethical and economic reasons, however, animal use has been heavily criticized and therefore became less acceptable. Hence, increasing attention is currently focused on the establishment and use of appropriate *in vitro *models, especially liver-based testing systems (EFPIA, 2012[8]). Among the *in vitro *liver models available today, primary human hepatocyte cultures are generally considered as the gold standard. Nevertheless, the overall shortage of human hepatocytes for research purposes, the restricted cultivation time due to the occurrence of dedifferentiation and the lack of cell dividing potential impede their general use. Immortalization of primary human hepatocytes, yielding a virtually unlimited cell supply and a fully functional hepatic cell line, could offer a solution (Deurholt et al., 2009[7]; Reid et al., 2009[20]). Over the years, a plethora of immortalized human hepatic cell lines has been introduced. A frequently applied strategy for the immortalization of adult human hepatocytes is based on the use of viral oncogenes, whether or not in combination with telomerase reverse transcriptase (TERT) protein. However, by using this methodology most hepatic cell lines often largely lack *in vivo*-like hepatic functionality (Ramboer et al., 2014[17]). 

In the present study, a novel immortalization strategy for hepatocytes is proposed. It relies on the observation that the p16-regulated premature growth arrest observed in several epithelial cells might be partially involved in the limited proliferation capacity of adult hepatocytes *in vitro* (Auer et al., 1998[1]; Tombes et al., 1998[26]; Ramirez et al., 2001[19], 2003[18]; Ohtani et al., 2004[15]; Harashima et al., 2013[9]; Ramboer et al., 2014[17]). This innovative immortalization technique has been successfully applied to human myogenic cells and uses a combination of 3 genes, namely cyclin D1, a mutant isoform of cyclin dependent kinase 4 (CDK4R24C) and TERT, in order to obtain cellular immortalization through abolishment of p16 function and reactivation of telomerase (Shiomi et al., 2011[24]). Here, we explore whether this strategy is also applicable to primary human hepatocytes. 

## Material and Methods

### Vector production and characterization 

Plasmids used for the production of the lentiviral vectors were kindly provided by Dr. T Kiyono (Shiomi et al., 2011[24]). The CMV-CDK4R24C, CMV-cyclin D1 and CMV-TERT lentiviral vectors were transfected with packaging vectors by a calcium phosphate transfection in human embryonic kidney 293t (HEK293t) cells. Supernatants, containing a viral construct, were collected and filtered 48 hours after transfection. The viral suspensions were stored at -80 °C. For characterization of the produced vectors, HEK293t cells were transduced with every single vector separately and samples for real time quantitative polymerase chain reaction (RT-qPCR) and western blot (WB) analyses were taken approximately 2 weeks after cultivation.

### Human hepatocyte cultivation and transduction

Cryopreserved human hepatocytes (donor No.HH223, BD bioscience, Belgium) were used in these experiments. Thawing was performed according to the manufacturer's instructions using the One step purification kit (BD Bioscience, Belgium). Human hepatocytes were seeded on a 24-well plastic surface at a density of approximately 2.10^5^ cells/well in Williams' medium E (Invitrogen, Belgium) supplemented with 7 ng/ml glucagon, 292 mg/ml L-glutamine, antibiotics (7.33 I.E./ml sodium benzyl penicillin, 50 µg/ml kanamycin monosulphate, 10 µg/ml sodium ampicillin, 50 µg/ml streptomycin sulphate) and 10 % v/v foetal bovine serum (Gibco, Belgium). Cell culture plates were placed in an incubator (37 °C, 5 % v/v CO_2_) and after approximately 16 hours, cell culture medium was removed and replaced by serum-rich medium supplemented with 25 µg/ml hydrocortisone sodium hemisuccinate and 0.5 µg/ml insulin ('growth cell culture medium'). On day 2 of cultivation, the cells were simultaneously exposed for approximately 16 hours to CMV-CDK4R24C, CMV-cyclin D1 and CMV-TERT lentiviral suspensions. All hepatocyte cultures were maintained in an incubator (37 °C, 5 % v/v CO_2_) and cell culture medium was replaced daily. 

### Hepatic cell line cultivation and differentiation

At 80 to 90 % confluence, cells were washed with phosphate-buffered saline (PBS) and trypisinized with TrypLE™ (Life technologies, Belgium). Cell counting was performed using a TC10™ automated cell counter (Bio-Rad, Belgium). Cells were either subcultured on a plastic surface in growth cell culture medium at a cell density of approximately 5500 cells/cm^2^ or frozen in growth cell culture medium supplemented with 10 % v/v dimethylsulfoxide. For differentiation purposes, cells were grown until nearly confluent in growth culture medium ('IHH-ET cells') and cultured for 2 more weeks in serum-free culture medium, composed of Williams' medium E supplemented with 7 ng/ml glucagon, 292 mg/ml L-glutamine, antibiotics (7.33 I.E./ml sodium benzyl penicillin, 50 µg/ml kanamycin monosulphate, 10 µg/ml sodium ampicillin, 50 µg/ml streptomycin sulphate), 25 µg/ml hydrocortisone sodium hemisuccinate and 0.5 µg/ml insulin ('differentiation cell culture medium'). All hepatocyte cultures were maintained in an incubator (37 °C, 5 % v/v CO_2_) and cell culture medium was replaced every 2 days.

### Real-time quantitative polymerase chain reaction analysis

Cells were washed with cold PBS and harvested by trypsinization. Total cellular ribonucleic acid (RNA) extraction, and complementary deoxyribonucleic acid (cDNA) production and cDNA purification were carried out as explained elsewhere (De Kock et al., 2012[6]). The RT-qPCR reaction mix and RT-qPCR conditions, using the StepOnePlus system (Applied Biosystems, Belgium), were established according to the manufacturer's instructions (Applied Biosystems, Belgium). Gene mixes were purchased from Applied Biosystems (Table 1(Tab. 1)). Selection of reliable housekeeping genes for normalization of the RT-qPCR data was done using geNorm within the qbase+ software (Biogazelle, Belgium). The results were processed with the qbase+ software (Biogazelle, Belgium).

### Western blot analysis

Cells were washed with cold PBS and harvested by trypsinization. Total cellular protein extraction was carried out using the radio-immunoprecipitation assay (RIPA) lysis buffer completed with Halt Protease Inhibitor Cocktail (Thermo Scientific Pierce, Belgium) and total cellular protein quantification was performed with the bichinchoninic acid protein kit and bovine serum albumin (BSA) as standard, both according to the manufacturer's instructions (Thermo Scientific Pierce, Belgium). Except for TERT analysis, proteins (25 μg) were heated, fractionated on sodium dodecyl sulphate polyacrylamide 7.5 % or 10 % w/v gel and blotted afterwards onto nitrocellulose membranes (Amersham, United Kingdom). To control the efficiency of the blotting as well as equal loading of the proteins on the gel, reversible protein staining was performed using a 0.1 % w/v Ponceau S solution. For TERT analysis, the subsequent steps were performed using the Supersignal Western Blot Enhancer kit (Pierce, Belgium). Membranes were blocked with 5 % w/v non-fatty milk in Tris-buffered saline solution (TBS-T). Membranes were incubated overnight at 4 °C with primary antibodies (Table 2(Tab. 2)) followed by incubation for 1 hour at room temperature with a horseradish peroxidase-conjugated secondary antibody (Dako, Denmark). Excess of antibody was removed by washing the membranes several times with TBS-T. Detection of the proteins was carried out by means of an enhanced chemiluminescence Western blotting system (Pierce Thermo Scientific, Belgium). Where applicable, blots were further incubated with a primary antibody directed against a reliable housekeeping gene (Table 2(Tab. 2)). 

### Population doubling time 

For doubling time assays, cells were plated in 6-well plates and cultured for 5 days in growth cell culture medium. At specific time points, namely after 6, 24, 48, 72, 96, 120 hours of cultivation, cells of 3 wells were washed once with 1.4 ml/well PBS, trypsinized with 1 ml/well TrypLE™ and resuspended in 0.250 ml growth cell culture medium. Cell numbers were determined by means of a TC10™ automated cell counter (Bio-Rad, Belgium). The population doubling time was calculated using the equation (t2-t1)/3.32 x (log n2- log n1), based on the linear part of the curve. 

### Flow cytometry

To determine the expression profile of the 3 immortalization genes in the whole cell population, flow cytometry analyses were performed. IHH-ET cells were harvested, fixed with 4 % w/v paraformaldehyde solution and permeabilized using a 0.1 % v/v PBS-Triton solution. After washing twice with PBS supplemented with 1 % w/v BSA, cells were incubated for 1 hour at room temperature with fluorescence labelled primary antibodies, namely CDK4-FITC, cyclin D1-R-PE and TERT-PerCP-Cy5.5. Following a final washing step, cells were resuspended in PBS, counterstained with Hoechst and analysed using the Attune^®^ Acoustic Focusing Cytometer (Life Technologies, Belgium). FITC, R-PE, PerCP-CY5.5 and Hoechst-positive events were recorded and the appropriate compensation settings were applied**.** Labelling of the primary antibodies, CDK4, cyclin D1 and TERT (Santa Cruz,USA), was carried out using the antibody concentration kit and conjugation kits (Abcam, UK).

### Light microscopy 

The morphology of the cell cultures was monitored every 2 days by inverse light microscopy (Nikon, Belgium). Images were taken at 100x magnification.

### Enzyme-linked immunosorbent assay

Cells were cultured in a 6-well plate and, following cultivation, the secretion of albumin (ALB) (AssayPro, USA) and alpha-1-antitrypsin (A1AT) (Abcam, UK) in the cell culture medium was quantified by enzyme-linked immunosorbent assay (ELISA) according to the manufacturer's instructions. For normalization of secretion values, the total protein content from each well was determined according to the Bradford method (Bradford, 1976[3]) using BSA as a standard.

### Glycogen storage

To demonstrate the presence of glycogen deposition, a Periodic acid Schiff (PAS) staining was performed. Briefly, cells were fixed with a 4 % w/v paraformaldehyde solution, washed 3 times with PBS and incubated with 100 mM glycin. After 3 additional washing steps, the cells were stained for 10 minutes with periodic acid, washed 3 times with PBS and then stained for 15 minutes with Schiff's reagent. Finally, cells were successively washed with PBS, counterstained with hematoxylin solution, rinsed with water and dried to air *prior* to microscopic examination and imaging at 200x magnification (Nikon, Belgium). To confirm glycogen accumulation and serving as a negative control, a 0.2 % w/v diastase digestion was completed before performing the PAS staining. 

### Statistical analysis

Results were evaluated using a 2-tailed unpaired Student's *t*-test with level of significance p ≤ 0.05. Data are expressed as mean ± standard deviation of cells originating from different cultivations (n) or from one and the same cultivation (N).

## Results

### Efficiency of the lentiviral vector constructs

Lentiviral vectors containing the coding sequences for cyclin D1, CDK4R24C and TERT were constructed. To test the efficiency of these vectors, HEK293t cells were transduced with every construct separately. As such, 4 different cell lines, namely 

(*i*) cyclin D1-transduced HEK293t cells, 

(*ii*) CDK4R24C-transduced HEK293t cells, 

(*iii*) TERT-transduced HEK293t cells and

(*iv*) non-transduced HEK293 cells ('control') were generated. 

**Figure 1:**
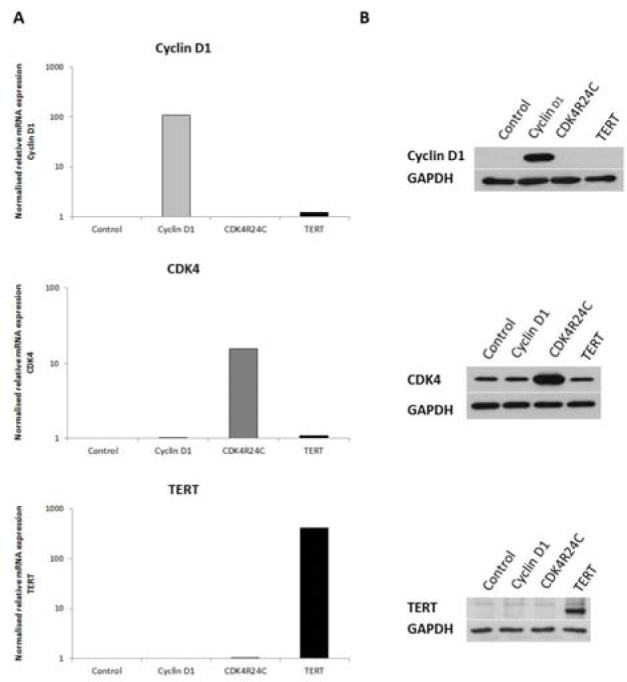
mRNA and protein expression levels of the immortalization genes in transduced HEK293t cells HEK293t cells were separately transduced with lentiviral vectors, coding for cyclin D1, CDK4R24C, and TERT. Samples were taken approximately 2 weeks after transduction (n=1, N=1). A) Samples were subjected to RT-qPCR analysis, using ACTB and GAPDH as a combination of reference genes for normalization. The results were processed with the qbase+ software and relative mRNA expression levels were normalized against the geometric mean of both reference genes and scaled to the values of non-transduced HEK293t cells. Each sample was analyzed in duplicate and results are expressed as mean in logarithmic scale. B) Samples (25 µg protein) were subjected to WB analysis. Each sample was analyzed in duplicate. Primary antibodies against cyclin D1, CDK4 or TERT were used followed by an appropriate secondary antibody. The protein expression of the endogenous control, GAPDH, is used as control for equal loading. (ACTB, beta actin; CDK4, cyclin-dependent kinase 4; GAPDH, glyceraldehyde 3-phosphate hydrogenase; HEK293t, human embryonic kidney 293t; mRNA, messenger ribonucleic acid; RT-qPCR, real time quantitative polymerase chain reaction; TERT, telomerase reverse transcriptase; WB, western blot)

The expression of the immortalization genes was determined at the transcriptional and translational level. Both mRNA and protein quantities of cyclin D1, CDK4 and TERT appear to be upregulated in the respective transduced HEK293t cell line compared to the non-transduced HEK293t cells (Figure 1(Fig. 1)).

### Development of a human hepatic cell line

**Figure 2:**
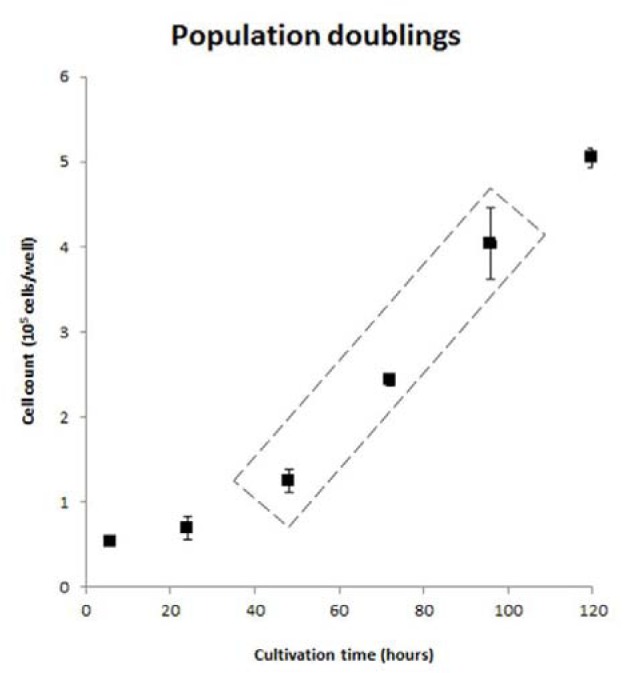
Population doublings of the cell line Cells were plated in 6-well plates and cultured for 5 days in growth cell culture medium. At specific time points, specifically after 6, 24, 48, 72, 96, 120 hours of cultivation, cell numbers from 3 different wells (N=3) were determined. Results are expressed as mean + standard deviation of 2 different cultivations (n=2). Boxed area indicates linear part of the curve used for calculation of PDT. (PDT, population doubling time)

Cryopreserved adult human hepatocytes were transduced with a combination of the 3 verified lentiviral vectors. Cell growth was observed within 2 weeks. The resulting cell line has been cultivated for over 15 passages and possesses a population doubling time (PDT) of approximately 30 hours (Figure 2(Fig. 2)). 

**Figure 3:**
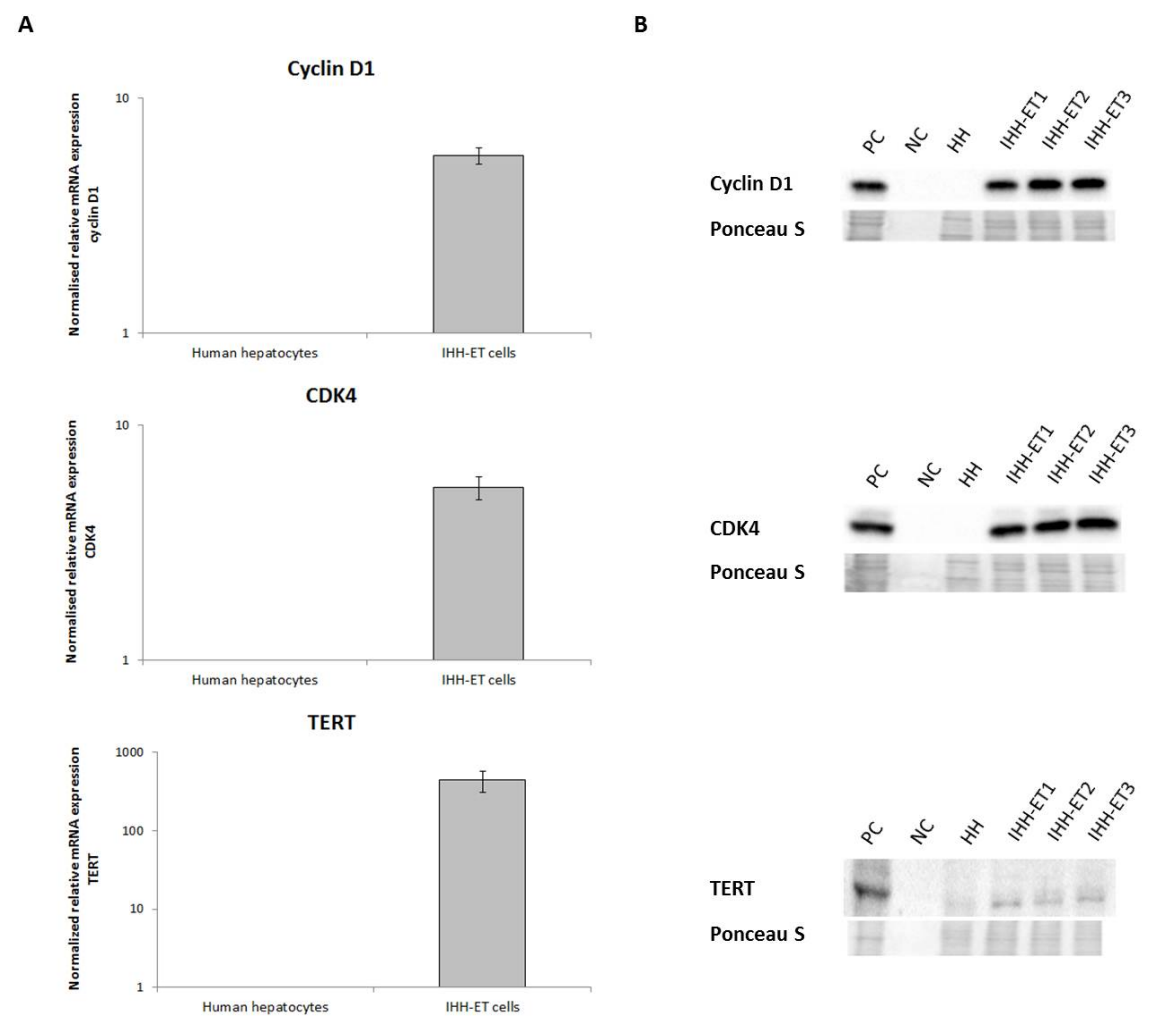
mRNA and protein expression levels of the immortalization genes in the cell line The cell line was grown in growth cell culture medium and sampling was performed upon 90 % confluence. Results represent IHH-ET cells of 3 different cultivations (n=3, N=1). The original primary human hepatocytes were included in the analyses as a reference (n=1, N=1). A) Samples were subjected to RT-qPCR analysis, using B2M, UBC, ACTB and HPRT1 as a combination of reference genes for normalization. Each sample was analyzed in duplicate. The results were processed with the qbase+ software and mRNA expression levels were normalized against the geometric mean of the selected reference genes and scaled to the values of original primary human hepatocytes. Results are expressed as mean + standard deviation in logarithmic scale. B) Samples (25 µg protein) were subjected to WB analysis. Primary antibodies against cyclin D1, CDK4 or TERT were used followed by appropriate secondary antibodies. Ponceau S staining is used as a control for equal loading and HEK293t cells (10 µg protein) transduced with the respective immortalization gene as positive control. Samples composed of RIPA lysis buffer and sample buffer without the addition of any cell lysate are used as negative control. (ACTB, beta actin; B2M, beta-2 microglobulin; CDK4, cyclin dependent kinase 4; HH, human hepatocytes; HPRT1, hypoxanthine ribosyltransferase 1; IHH-ET, immortalized human hepatocytes; mRNA, messenger ribonucleic acid; NC, negative control; PC, positive control; RIPA, radio-immunoprecipitation assay; RT-qPCR, real time quantitative polymerase chain reaction; TERT, telomerase reverse transcriptase; UBC, ubiquitin C; WB, western blot)

RT-qPCR (Figure 3A(Fig. 3)) and WB (Figure 3B(Fig. 3)) analyses revealed apparently increased levels of cyclin D1, CDK4 and TERT in the transduced cell line compared to the non-transduced cells. 

**Figure 4:**
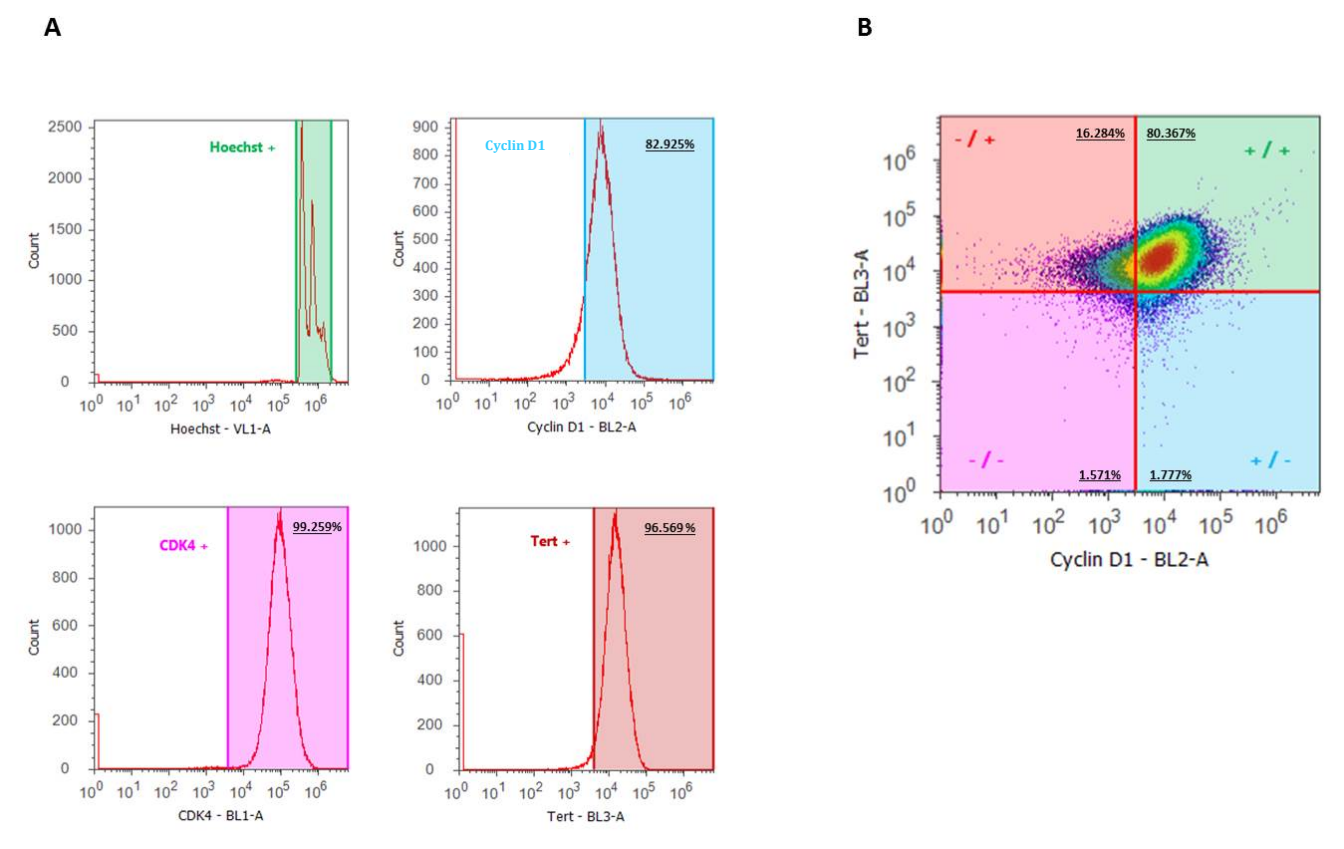
Flow cytometry analysis of immortalization gene expression profiles The cell line was grown in growth cell culture medium and cells were trypsinized at 90 % confluence, fixed and labeled with fluorescence labeled primary antibodies. Results represent IHH-ET cells from one cultivation (n=1, N=1). (A) The cell line was selected and tested for CDK4-FITC, cyclin D1-R-PE, TERT-PerCP-Cy5.5 and Hoechst-positive events. The number of positive events in the selected population is indicated as percentage on the graph. (B) The combined expression of cyclin D1 and TERT in the selected cell line population is determined. The right upper side represents the dual cyclin D1 and TERT-positive events in the selected cell line population. The number of events is indicated as a percentage in every quarter of the graph. (CDK4, cyclin dependent kinase 4; Cy, cyanine; FITC, fluorescein; FSC, forward scatter; IHH-ET cells, immortalized human hepatocytes; PerCP, Peridinin chlorophyll protein complex; R-PE, R-phycoerythrin; TERT, telomerase reverse transcriptase; SSC, side scatter)

To determine the expression profile of the 3 immortalization genes in the whole cell line population, flow cytometry analyses were carried out. It was found that CDK4, TERT and cyclin D1 were expressed by 99.3 %, 96.6 % and 82.9 % of the cells, respectively (Figure 4A(Fig. 4)). Combination of the expression profiles of cyclin D1 and TERT indicates that at least 80.4 % of the cells possess the 3 immortalization genes (Figure 4B(Fig. 4)). 

### Morphological and functional characterization of a human hepatic cell line

**Figure 5:**
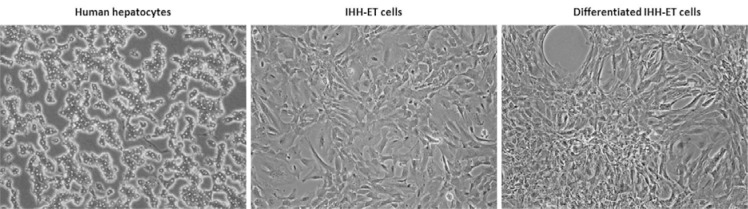
Morphologic appearance of the cell line The cell line was cultured in growth cell culture medium until nearly confluent (IHH-ET cells) or 2 more weeks in differentiation cell culture medium (DIHH-ET cells). Images were taken at 100x magnification. The figure is representative for IHH-ET and DIHH-ET cells from 3 different cultivations (n=3). A figure of primary human hepatocytes is included for comparison. (DIHH-ET, differentiated immortalized human hepatocytes; IHH-ET, immortalized human hepatocytes)

To characterize the newly developed cell line, the morphology of the cells was monitored during cultivation. As depicted in Figure 5(Fig. 5), the cells do not possess the typical cuboidal morphology of human hepatocytes. Structural heterogeneity can be observed within the transduced cell population. When the cells were cultured for 2 more weeks in differentiation cell culture medium to trigger differentiation, cellular overgrowth occurred and cells started to detach from the plastic surface. However, acquisition of the hepatocyte-specific morphology remained absent. 

**Figure 6:**
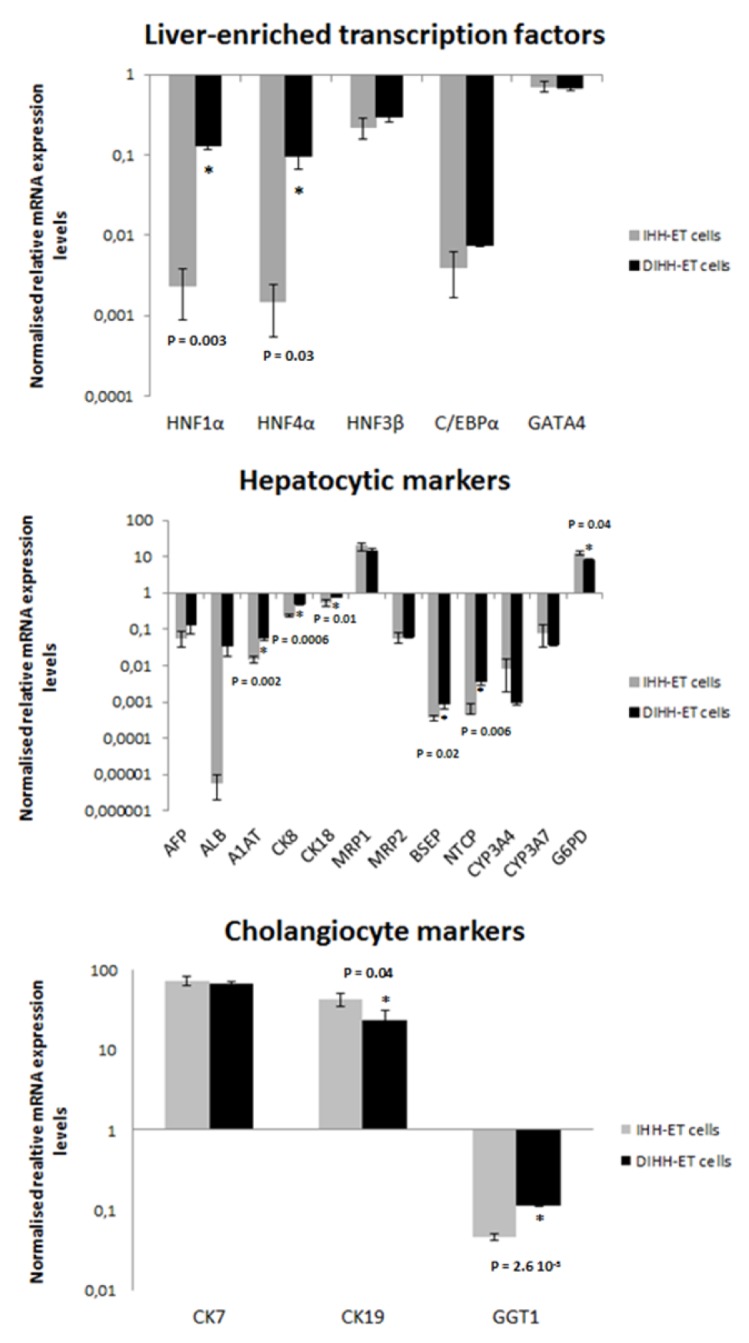
mRNA expression levels of liver-specific markers in the cell line The cell line was cultured in growth cell culture medium until nearly confluent (IHH-ET cells) or 2 more weeks in differentiation cell culture medium (DIHH-ET cells) before samples were taken. Samples were subjected to RT-qPCR analysis, using B2M, UBC, ACTB and HPRT1 as a combination of reference genes for normalization. Samples were analyzed in duplicate. The results were processed with the qbase+ software and mRNA expression levels were normalized against the geometric mean of the selected reference genes and scaled to the original primary human hepatocytes. Results are expressed as mean + standard deviation in logarithmic scale and represent IHH-ET and DIHH-ET cells of 3 different cultivations (n=3, N=1). Statistical analysis was performed using a 2-tailed unpaired *t*-test with significance p < 0.05 (*). (A1AT, alpha-1-antitrypsin; ACTB, beta actin; AFP, alpha-fetoprotein; ALB, albumin; B2M, beta-2 microglobulin; BSEP, bile salt export pump; C/EBP, CCAAT/enhancer binding protein; CK, cytokeratin; CYP, cytochrome P450; DIHH-ET, differentiated IHH-ET; G6PD, glucose-6-phosphatase dehydrogenase; GGT1, gamma-glutamyltransferase 1; HNF, hepatocyte nuclear factor; HPRT1, hypoxanthine ribosyltransferase 1; IHH-ET, immortalized human hepatocytes; mRNA, messenger ribonucleic acid; MRP, multidrug resistance-associated protein; NTCP, sodium-dependent taurocholate cotransporting polypeptide; RT-qPCR, real time quantitative polymerase chain reaction; UBC, ubiquitin C)

**Figure 7:**
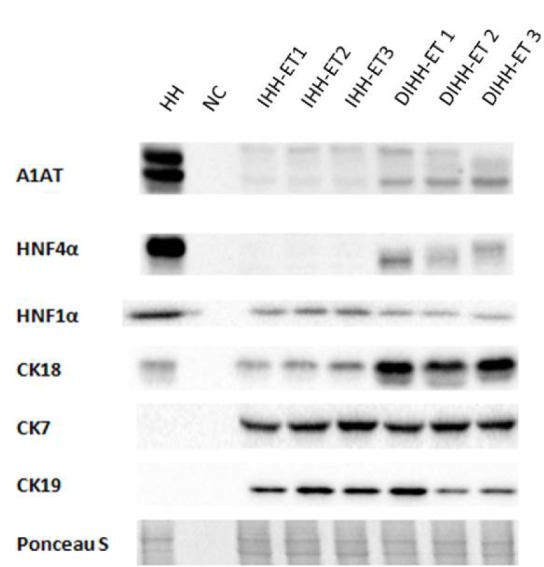
Protein expression levels of liver-specific markers in the cell line The cell line was cultured in growth cell culture medium until nearly confluent (IHH-ET cells) or 2 more weeks in differentiation cell culture medium (DIHH-ET cells) before samples were taken. Samples (25 µg protein) were subjected to WB analysis. Primary antibodies directed against respective antigens were used followed by an appropriate secondary antibody. Ponceau S staining is used as a control for equal loading. Results represent cells of 3 different cultivations (n=3, N=1). Samples composed of RIPA lysis buffer and sample buffer without the addition of any cell lysate are used as negative controls. (A1AT, alpha-1-antitrypsin; CK, cytokeratin; DIHH-ET, differentiated IHH-ET; HH, human hepatocytes; HNF, hepatocyte nuclear factor; IHH-ET, immortalized human hepatocytes; NC, negative control: WB, Western blot)

Hepatic functionality of the cell line was tested using different techniques. First, the expression of multiple liver-specific markers was examined by RT-qPCR and WB analyses (Figures 6(Fig. 6) and 7(Fig. 7)). It was found that the mRNA levels of most liver-enriched transcription factors,* in casu *hepatocyte nuclear factor (HNF)1α, HNF4α, HNF3β and CCAAT/enhancer binding protein α (C/EBPα), as well as liver-specific proteins, namely ALB, A1AT and alpha-fetoprotein (AFP) were low in IHH-ET cells. Upon differentiation, mRNA quantities of A1AT, HNF1α and HNF4α became significantly increased, but were still lower than the values observed in non-transduced primary hepatocytes. The same trend was observed at the protein level for HNF4α and A1AT, but not for HNF1α. The mRNA levels of the metabolizing enzyme cytochrome P450 monooxygenase (CYP) 3A4 and its fetal counterpart CYP3A7, the uptake transporter sodium-dependent taurocholate cotransporting polypeptide (NTCP) and the efflux transporters multidrug resistance-associated protein (MRP) 2 and bile salt export pump (BSEP), were also downregulated in IHH-ET cells. Only NTCP and BSEP mRNA quantities were elevated after differentiation. However, the levels reached were still very low compared to those observed in non-transduced primary hepatocytes. Both IHH-ET and differentiated IHH-ET (DIHH-ET) cells did not longer express the uptake transporter organic anion transporting polypeptide 1B1. In contrast, MRP1, which is barely expressed in adult human hepatocytes (Ct-value=33), was clearly present in the established cell line. Similar changes were noticed for the mature hepatocyte marker glucose-6-phosphate dehydrogenase (G6PD). When analyzing the mRNA expression of CYP1A2, very low expression levels were detected in the non-transduced human hepatocytes (Ct-value=33). With regard to cytokeratins (CKs), IHH-ET cells displayed an apparent decrease in CK8 and CK18 mRNA abundance. However, for both CKs, an increase in mRNA amounts was observed in the DIHH-ET cells, which was also visible for CK18 at the protein level. Furthermore, IHH-ET cells tended to express high mRNA and protein amounts of CK7 and CK19, which are cholangiocyte markers found at insignificant mRNA (Ct-value=34) and protein levels in the non-transduced human hepatocytes. In contrast to other CKs, the mRNA levels of CK19 were decreased in DIHH-ET cells, which was equally visible at the protein level for DIHH-ET2 and DIHH-ET3 cells. Another biliary marker, gamma-glutamyltransferase 1 (GGT1) was detected in the non-transduced human hepatocytes (Ct-value=24) and exhibited an opposite regulation to CK19 in IHH-ET and DIHH-ET cells. 

**Figure 8:**
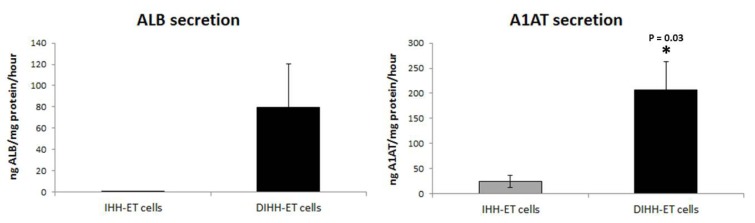
Secretion of liver-specific proteins of the cell line Cells were grown in 6-well plates and secretion of ALB and A1AT in cell culture medium of 3 different wells (N=3) was measured by means of ELISA. Results are expressed as mean + standard deviation of 3 different cultivations (n=3).Statistical analysis was performed using a 2-tailed unpaired *t*-test with significance p < 0.05 (*). (A1AT, alpha-1-antitrypsin; ALB, albumin; DIHH-ET cells, differentiated IHH-ET cells; ELISA, enzyme-linked immunosorbent assay; IHH-ET cells, immortalized human hepatocytes)

In accordance with the RT-qPCR data, the amount of A1AT and ALB secreted *per* hour in the culture medium for the IHH-ET cells was very low (25 ± 12 ng A1AT/mg protein) or even hardly measurable (8 10^-4 ^± 4 10^-4^ ng ALB/mg protein), but reached higher levels (207 ± 56 ng A1AT/mg protein; 79 ± 41 ng ALB/mg protein) in the DIHH-ET cells (Figure 8(Fig. 8)). 

**Figure 9:**
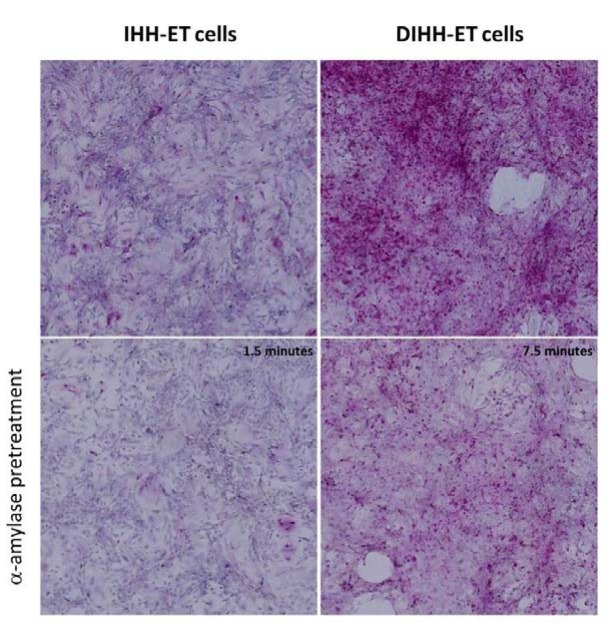
Glycogen storage in the cell line Cells were grown in 6-well plates and after cultivation a PAS-staining was performed. Diastase treatment served as negative control with IHH-ET and DIHH-ET cells incubated for 1.5 and 7.5 minutes, respectively. Images were taken at 200x magnification. Images are representative for cells of 3 different cultivations (n=3). (DIHH-ET, differentiated IHH-ET; IHH-ET, immortalized human hepatocytes, PAS, Periodic acid Schiff)

With respect to the capacity for glycogen storage, a difference can be observed between the IHH-ET and DIHH-ET cells. Only differentiated cells displayed a clear PAS-staining, which visibly decreased after α-amylase treatment (Figure 9(Fig. 9)).

## Discussion

The aim of this study was to develop a human hepatic cell line using an established immortalization strategy (Shiomi et al., 2011[24]), based on the combined overexpression of TERT and the cell cycle regulators, cyclin D1 and CDK4R24C, rather than viral oncogenes to overcome the early growth arrest of adult human hepatocytes and immortalize the cells. When adult human hepatocytes were simultaneously transduced with the 3 immortalization genes, colony formation became visible in the culture system and a cell line was established. The proliferating cells possessed a PDT of approximately 30 hours, which is in line with those of other immortalized human hepatic cell lines (Pfeifer et al., 1993[16]; Nguyen et al., 2005[14]). However, as hepatocyte differentiation and proliferation are typically mutually exclusive *in vitro, *cell growth was associated with deterioration of the differentiated morphology and functional phenotype, a phenomenon already described by others (Kim et al., 2000[11]; Chamuleau et al., 2005[4]). It was found that IHH-ET cells display downregulated expression of most hepatocytic markers and liver-enriched transcription factors at the transcriptional and/or translational level. In contrast, mRNA quantities of MRP1 were elevated. The expression of this efflux drug transporter has been linked to the proliferation rate of hepatocytes, with increased mRNA and protein levels in proliferating hepatocytes (Roelofsen et al., 1997[22], 1999[21]; Schippers et al., 1997[23]). Upregulation of the mRNA production was also observed for G6PD, a mature hepatocyte marker. Its deviant expression pattern in proliferating hepatocytes remains elusive, yet enhanced activity of this marker has been associated with cellular proliferation in NIH 3T3 cells (Kuo and Tang, 1999[13]). Besides the observed changes for hepatocytic markers, the IHH-ET cells expressed the cholangiocyte markers CK7 and CK19, which may point to hepatocyte dedifferentiation (Blaheta et al., 1998[2]; Deurholt et al., 2009[7]). The appearance of a mixed CK phenotype in immortalized hepatic cell lines has previously been described (Smalley et al., 2001[25]; Clayton et al., 2005[5]). The concomitant increased expression of the hepatic progenitor cell marker THY1 (data not shown), actually suggests that the IHH-ET cells possess a rather bipotential hepatic progenitor-like phenotype (Iacob et al., 2011[10]). 

Over the years, several strategies to counteract the dedifferentiation of hepatic cell lines have been proposed. The use of reversible immortalization techniques has proven useful to some extent and might be interesting to introduce in future cell lines (Kobayashi et al., 2000[12]; Chamuleau et al., 2005[4]; Nguyen et al., 2005[14]; Totsugawa et al., 2007[27]). Furthermore, increasing attention should be paid to culture systems that support and/or promote the differentiation status of immortalized hepatocytes, such as 3D or co-culture systems (Werner et al., 1999[29], 2000[30]; Watanabe et al., 2003[28]; Chamuleau et al., 2005[4]; Deurholt et al., 2009[7]; Zhao et al., 2012[31]). In this study, cellular overgrowth occurred when IHH-ET cells were cultivated for 2 more weeks in serum-free culture medium. While no improvement was observed morphologically, the cell line displayed an enhanced hepatic phenotype. Indeed, increased expression of several hepatocytic markers, including A1AT, CK8, CK18, NTCP and BSEP and the liver-enriched transcription factors, HNF1α and HNF4α was detected at the transcriptional and/or translational level. Moreover, DIHH-ET cells secreted higher levels of the liver-specific proteins ALB and A1AT and displayed glycogen deposition. 

In conclusion, the innovative immortalization strategy applied to primary human hepatocytes in this study generates a novel hepatic cell line that seems to retain some key hepatic characteristics when cultured under appropriate conditions. 

## Acknowledgements

This work was financially supported by the grants of the University Hospital of the Vrije Universiteit Brussel-Belgium (Willy Gepts Fonds UZ-VUB), the Fund for Scientific Research-Flanders (FWO grants G009514N and G010214N) and the European Research Council (ERC Starting Grant 335476). The authors thank Dr. T. Kiyono for providing the vector plasmids and the VIB unit of molecular and cellular oncology under supervision of Prof. G. Berx for their scientific guidance and technical assistance with the lentiviral vector and hepatic cell line production. Furthermore, the authors also want to thank Tineke Vanhalewyn for the technical assistance with the hepatic cell line cultivation.

## Conflict of interest

The authors declare that they have no conflict of interest. 

## Figures and Tables

**Table 1:**
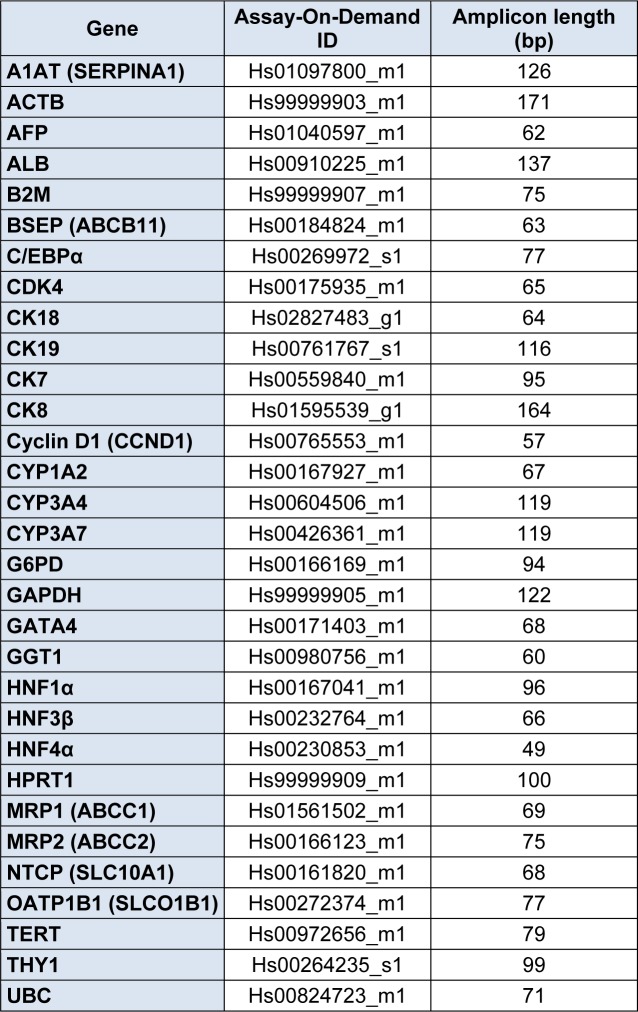
Gene expression assays used for real time quantitative polymerase chain reaction analysis

**Table 2:**
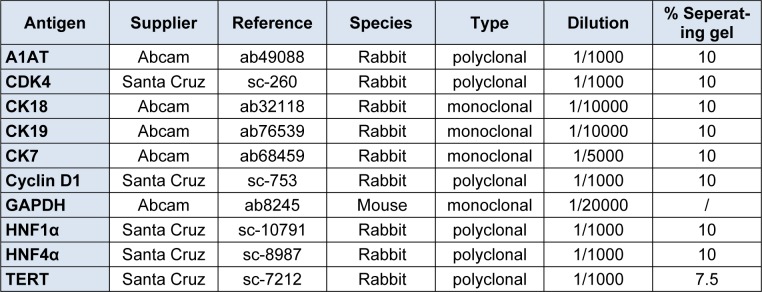
Primary antibodies used for western blot analysis
